# Pathogenesis of the *Pseudomonas aeruginosa* Biofilm: A Review

**DOI:** 10.3390/pathogens11030300

**Published:** 2022-02-27

**Authors:** Felipe Francisco Tuon, Leticia Ramos Dantas, Paula Hansen Suss, Victoria Stadler Tasca Ribeiro

**Affiliations:** Laboratory of Emerging Infectious Diseases, School of Medicine, Pontifícia Universidade Católica do Paraná, Curitiba 80215-901, Brazil; leticia.dantas@pucpr.br (L.R.D.); paula.h@pucpr.br (P.H.S.); vicstadler@gmail.com (V.S.T.R.)

**Keywords:** *Pseudomonas aeruginosa*, biofilm, quorum sensing, virulence

## Abstract

*Pseudomonas aeruginosa* is associated with several human infections, mainly related to healthcare services. In the hospital, it is associated with resistance to several antibiotics, which poses a great challenge to therapy. However, one of the biggest challenges in treating *P. aeruginosa* infections is that related to biofilms. The complex structure of the *P. aeruginosa* biofilm contributes an additional factor to the pathogenicity of this microorganism, leading to therapeutic failure, in addition to escape from the immune system, and generating chronic infections that are difficult to eradicate. In this review, we address several molecular aspects of the pathogenicity of *P. aeruginosa* biofilms.

## 1. Introduction

Biofilms are the underlying cause of a variety of tissue- and implant-associated infections. Infections associated with biofilms encompass tooth caries, periodontitis, otitis media, chronic sinusitis, chronic wound changes, musculoskeletal infections (osteomyelitis), biliary tract infection, bacterial prostatitis, native valve endocarditis, and medical device-related infections. *Pseudomonas aeruginosa* is considered a model organism for studying biofilm formation and is the most studied microorganism with regard to quorum sensing (QS). In this section, we describe the pathogenicity of the *P. aeruginosa* biofilm, including its characteristics and QS properties.

## 2. *Pseudomonas* *aeruginosa* Characteristics

*Pseudomonas aeruginosa* is a Gram-negative aerobic rod-shaped bacterium that can be isolated from most environments, including soil, plants, and mammal tissue [1]. This bacterium can survive on water, different surfaces, and medical devices using its influential binding factors, such as flagella, pili, and biofilms. Hence, *P. aeruginosa* is abundant in natural and artificial environments, including lakes, hospitals, and household sink drains [2].

**Figure 1:**
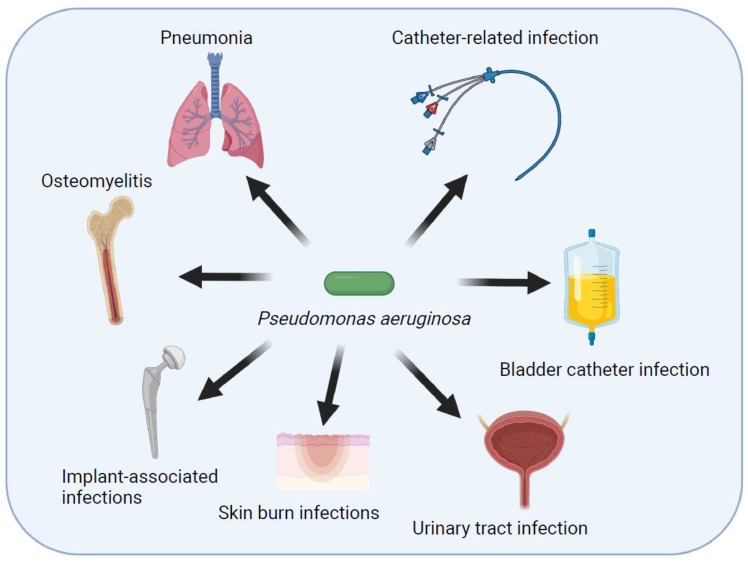
Schematic representation of main infections caused by *Pseudomonas aeruginosa*.

*Pseudomonas aeruginosa* is an opportunistic pathogen that causes several infections in humans (Figure 1). It has become an important cause of nosocomial infections and antibiotic resistance [3,4]. *Pseudomonas aeruginosa* can be specified as one of the opportunistic bacteria related to healthcare infections, including ventilator-associated pneumonia (VAP), intensive care unit infections, central line-related blood stream infections, surgical site infections, urinary tract infections, burn wound infections, keratitis, and otitis media [5,6,7,8]. *Pseudomonas aeruginosa* is an organism capable of adapting to changes in the environment, rapidly developing resistance to antibiotics, and producing a variety of virulence factors.

This pathogen can affect immunocompromised patients, in part due to its ability to evade both innate and acquired immune defenses through adhesion, colonization, and biofilm formation and to produce various virulence factors that cause significant tissue damage [9]. It also causes diseases with a high mortality rate in patients diagnosed with cystic fibrosis, neonatal infections, cancer, and severe burns [9,10].

Infections caused by *P. aeruginosa* can be life-threatening when inadequate therapy is employed, particularly when multidrug-resistant (MDR) strains are involved [11,12,13,14]. Multidrug resistance has been a threat to human and animal health for the last 30 years. Furthermore, *P. aeruginosa* is one of the most prevalent pathogens in hospital environments, causing more than 50% of healthcare-acquired infections [15]. Even though new antimicrobial drugs have been developed, the mortality rates due to *P. aeruginosa* remain high, in the range of 20–60% [5,8,12,13,14,16].

The major risk factors for *P. aeruginosa* infections are structural lung diseases, hematological neoplasms, transplantation, skin burns, recent use of antibiotics, presence of implants, prolonged hospitalization, and mechanical ventilation [17].

Considering the pathogenesis of *P. aeruginosa* infections, biofilm production is one of the most important virulence determinants. *Pseudomonas aeruginosa* is a well-known biofilm producer, making it an interesting in vitro model to understand biofilm formation [18]. *Pseudomonas aeruginosa* also colonizes several different surfaces, including medical materials and food industry equipment, and form biofilms, leading to chronic infections owing to increased resistance to antibiotics, various irradiation treatments, environmental conditions, disinfectants, and the immune system [19,20].

## 3. *Pseudomonas aeruginosa* Biofilms

Two distinct lifestyles, planktonic and sessile cells, can be adopted by *P. aeruginosa*. The planktonic state can be encountered in a liquid culture suspension, whereas on natural or synthetic surfaces, *P. aeruginosa* can form sticky clusters in permanent rearrangements characterized by the secretion of an adhesive and protective matrix [21]. Defined as a “biofilm,” this bacterial community adhering to a surface appears as an adaptive response to an environment more or less unsuited to growth in planktonic form [22].

Biofilms are dense aggregations of cells that produce extracellular matrix components that hold the community together. The biofilm mode of growth allows cells to stay close to nutrients, promotes the exchange of genetic material, and protects cells from a variety of chemical and environmental stresses, including engulfment by phagocytes [23]. A biofilm is composed of a self-secreted matrix made up of proteins (<2%), DNA (<1%), polysaccharides (1–2%), and RNA (<1%), with water as the remainder (97%). Alginate, Psl, and Pel are the three exopolysaccharides responsible for the main components in the biofilm matrix. They carry out several biological functions, especially with respect to the protection of the bacterial cell from antibiotics and the human immune system [24].

Bacterial biofilm has been evaluated for exhibiting phenotypic heterogeneity due to chemical variation within the biofilm, including oxygen gradients [25], nutrient fluctuations [26], and pH changes [27]. These environmental conditions are detected by individual bacterial cells, leading to metabolic activity and differential gene expression, even within a genetically homogeneous population [28]. Biofilms are approximately 10–1000 times more resistant to antibiotics than planktonic cells. This is due to the lack of antibiotic penetration into the complex polysaccharide matrix, as well as modification of the metabolic activity and protein synthesis [29,30].

Bacteria that grow in biofilms often exhibit a variety of phenotypic differences compared with the same strains grown in planktonic culture. These differences include changes in mobility, in some cases increases in the production of extracellular polysaccharides, and increased resistance to antibiotics [31,32]. Studies using DNA microarray analysis have revealed differences in the expression of more than 70 genes of *P. aeruginosa* following five days of biofilm growth [33]. Proteomic analysis revealed an almost 50% change in protein profiles following attachment of *P. aeruginosa* to an inorganic surface before developing a biofilm [34].

Considering all these aspects, for infections caused by biofilm-forming *P. aeruginosa*, such as in cystic fibrosis, the eradication of the pathogen is almost impossible, and additional challenges are encountered when treating infections caused by MDR strains [35]. These complications increase patient morbidity and mortality. Higher costs of treatment as well as greater frequencies and longer periods of hospitalization are other outcomes [19,36]. Macrophages exposed to biofilms exhibit secretory properties, and thus transform into cells responsible for tissue injury, even without direct contact with bacteria [37].

A greater understanding of the biofilm structure and composition, including the molecular mechanisms, and antimicrobial tolerance within the biofilm is important for the design of strategies to prevent, manage, and eradicate biofilm-associated infections.

## 4. Biofilm Structure and Dynamics

Alginate is an exopolysaccharide and one of the major components of the biofilm in mucoid strains of *P. aeruginosa*. The high molecular weight of this molecule is composed of d-mannuronic and l-guluronic acids, β1-4 linked and O-acetylated. These enzymes are coded by algD operon for alginate synthesis; their expression is regulated by AlgT σ-factor [38]. Alginate is overproduced by mucoid *P. aeruginosa* strains, and despite not being necessary for biofilm formation, is important in its maturation and stability [18,39,40].

Alginate attaches to mucin found in the respiratory tract, acting as an adhesin, and its acetyl groups increase the viscosity, which accumulate water and nutrients in the biofilm [38]. Alginate also contributes to persistence of the bacterium by protecting *P. aeruginosa* against phagocytosis and scavenging radical oxygen species released by activated macrophages [41]. Furthermore, alginate elicit a strong leukocyte response, the release of radical oxygen species, which contributes to lung inflammation [42]. Another important property of alginate is its ability to bind aminoglycoside antibiotics, impairing their penetration into the biofilm, which enhances the resistance against antibiotics as well as clinical therapeutic failure [43].

Rhamnolipids are amphipathic molecules found in the extracellular environment. They are secondary metabolites formed by a rhamnose linked via a dimer of a β-hydroxy fatty acid bond to O-glycosidic. These molecules have the ability to degrade the lung surfactant [44], decreasing the transepithelial electrical resistance, leading to the disruption of tight junctions of respiratory cells [45,46].

**Figure 2:**
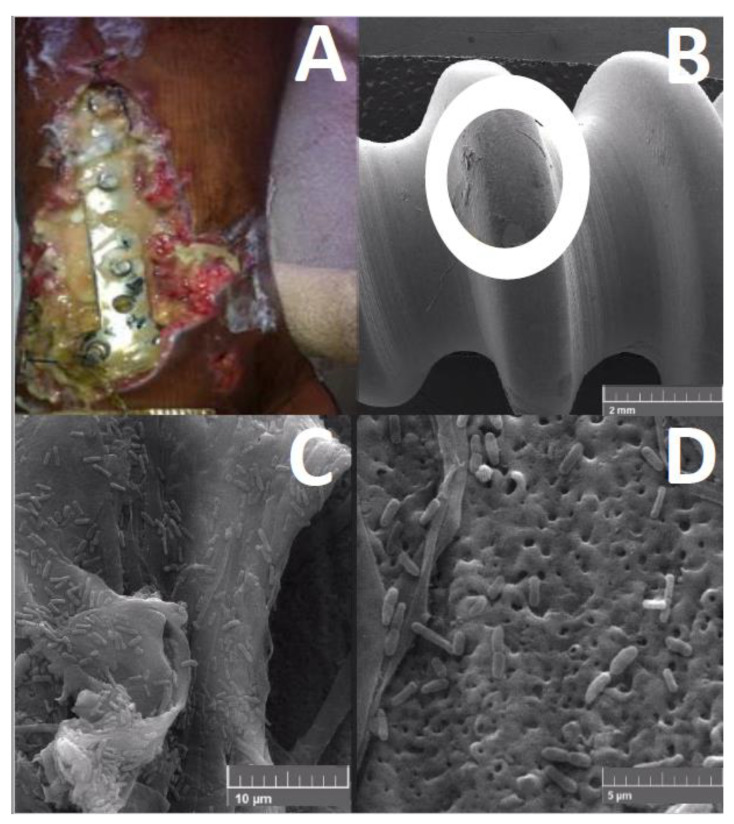
Biofilm formed by *Pseudomonas aeruginosa* on an orthopedical device. (**A**)—Clinical infection after exposure tibial fracture; (**B**)—Biofilm identification using scanning electronic microscopy in the orthopedic screw of the same patient; (**C**,**D**)—*P. aeruginosa* biofilm in the surgical screw under greater magnifications. Source: authors.

This condition promotes the colonization of the airways by *P. aeruginosa*, increasing the chance of pneumonia [47]. Rhamnolipids contribute to biofilm architecture by keeping the non-colonized channels open [48] (see Figure 2D). Rhamnolipids facilitate sliding motility in the absence of flagella and enable swarming motility by decreasing the surface tension, owing to their surfactant properties [49,50,51]. Iron-restricted conditions induce the production of rhamnolipids, promoting twitching motility [52]. Host innate immunity can be suppressed by blocking flagellin-induced human β-defensin 2 [53].

## 5. Regulation Systems Involved in *Pseudomonas aeruginosa* Biofilm Formation

The communication of bacterial cells has been shown to be important for the development of *P. aeruginosa* biofilms [54]. The virulence genes in *P. aeruginosa* are modulated by a bacterial cell-to-cell signaling mechanism, known as QS [55]. QS bacteria produce small signaling molecules that at a high population density can interact with associated receptors. This interaction induces the expression of various genes related to biofilm production and bacterial virulence [55]. QS plays a pivotal role in the pathogenesis of *P. aeruginosa*, beginning with host colonization to invasion, infection, dissemination, immune evasion, and drug resistance [56].

The four most important interconnected systems of QS in *P. aeruginosa* are termed Las, Rhl, Pqs, and Iqs. These hierarchical network systems enable crosstalk between multiple cellular signals through QS. The LasR/LasI and RhlR/RhlI QS systems regulate synthesis and signal transduction via 3-oxo-C12-HSL and C4-HSL, respectively. LasI synthesizes 3-oxoC12-HSL, which activates the cytoplasmic receptor. LasR regulates the expression of hemolysins, proteases, elastases, and exotoxin-A production genes for biofilm formation [57]. LasR in *P. aeruginosa* was identified to be a key regulator in the expression of the lasB gene, which encodes for a metalloprotease elastase [58]. Subsequently, LasR was shown to be necessary for the transcription of *aprA*, *lasA*, and *toxA*. Hence, it is considered a global regulator of the virulence genes in *P. aeruginosa* [59].

The chemical structure of this gene-mediated *Pseudomonas* autoinducer (PAI) was recently characterized as that of N-(3-oxododecanoyl)-homoserine lactone (OdDHL) [60]. In *P. aeruginosa*, the LasR protein is required for the activation of *lasB* and several other virulence genes. The PAI produced by the bacteria is required for the activity of LasR. Another autoinducer, structurally identified to be N-butyrylhomoserine lactone (BHL), has been discovered [61]. BHL does not interact with LasR protein directly to activate *lasB* gene expression [60]. RhlR, a regulatory protein encoded by the rhamnolipid synthase gene cluster *rhlABR*, is the cognate receptor of BHL [62].

The third QS signal, 2-heptyl-3-hydroxy-4-quinolone, also called Pseudomonas quinolone signal (PQS), is associated with *lasB* expression in a lasR mutant of *P. aeruginosa*, and cannot be mimicked by OdDHL or BHL [63].

The fourth intercellular communication signal named as Iqs belongs to a new class of QS signal molecules. The genes involved in Iqs synthesis form a non-ribosomal peptide synthase gene cluster, ambBCDE [64]. When disrupted, it causes a decrease in the production of PQS as well as BHL signals. The same pathway induces the expression of virulence factors such as pyocyanin, rhamnolipids, and elastase.

**Figure 3:**
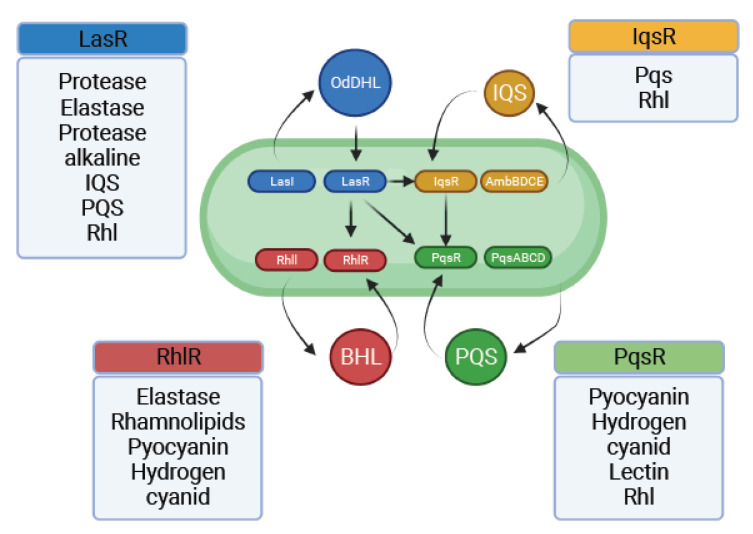
Genes of the quorum sensing systems of Pseudomonas aeruginosa with regulatory looping and molecules associated with biofilms and pathogenicity.

The QS circuits in *P. aeruginosa* are complex. The Las system, activated by OdDHL, promotes LasR-OdDHL complex multimerization and consequently activates the transcription of *rhlR*, *rhlI*, *lasI*, and other virulence genes [65,66,67]. The RhlR-BHL complex also activates the expression of its own regulon and RhlI, forming a second positive feedback loop. LasR-OdDHL also positively regulates PqsR, the transcriptional regulator of the PQS biosynthesis operon *pqsABCD* [68]. PQS, in turn, was found to be able to enhance the transcription of RhlI, thus influencing BHL production and the overall expression of the Rhl QS system and indirectly modulating the Rhl-dependent phenotypes [69] (Figure 3).

*Pseudomonas aeruginosa* invades hosts with a local or systemic immune system deficit or attenuated epithelial barrier in a passive manner. However, an outer-membrane protein, OprF, can recognize and bind to IFN-γ, activating the Rhl QS system. This pathway enhances the expression of lecA and the production of its encoded virulence protein, galactophilic lectin. Pyocyanin is another virulence factor that is upregulated in the presence of IFN-γ [70]. Human dynorphin is an endogenous κ-receptor agonist that can penetrate the bacterial membrane and potentially induce the expression of PqsR and PqsABCDE, increasing the biosynthesis of PQS and HHQ [71].

C-type natriuretic peptide (CNP), which is produced by endothelial cells, is also involved in the regulation of *P. aeruginosa* QS, inducing a rise in intracellular cAMP concentrations, which leads to the activation of the global virulence activator Vfr [72].

The stationary-phase sigma factor RpoS is involved in the control of biofilm growth in *P. aeruginosa* [73]. Approximately 700 genes, most of them related to QS, are under direct or indirect control of RpoS [74]. Bouillet et al. (2019) demonstrated that the post-translational regulation of RpoS depends on the HsbR-HsbA partner-switch. This system is connected to the FlgM-HsbA partner-switch, triggering the release or sequestration of RpoS. This mechanism is likely the most efficient system this bacterium has found to decide to swim or to form and disperse its biofilm [75].

More recently, PQS has been implicated in the first stages of biofilm development. It functions as an outer membrane vesicle (OMV) inducer that contributes at multiple stages. It has been demonstrated that PQS and OMVs are differentially produced during *P. aeruginosa* biofilm development, providing evidence that effective biofilm dispersion is dependent on the production of PQS-induced OMVs [76]. It is interesting that these genes involved in QS constitute nearly 10% of the *P. aeruginosa* genome and therefore account for a majority of the physiological processes and virulence phenotypes [77].

## 6. Mechanisms of Virulence in *Pseudomonas aeruginosa*

In general, *P. aeruginosa* adopts a sessile lifestyle during chronic infections or a planktonic lifestyle for acute infections. Several other mechanisms of virulence in *P. aeruginosa*, including motility (flagella and pili), immune evasion (elastase and alkaline protease), antibiotic resistance (pump efflux and modifying enzymes), cytotoxicity (hydrogen cyanide (HCN), exotoxin A, T3SS, and pyocyanin), iron scavenging (proteases and siderophores), and finally biofilm structure and dynamics (alginate and rhamnolipids), have been described [78] (Table 1).

*Pseudomonas aeruginosa* encodes virulence factors that increase its fitness and chances of survival within a human host. These virulence factors promote bacterial growth and survival, maneuvering the host cellular machinery by causing devastating injuries, tissue necrosis, evasion, and immune system impairment [79].

## 7. Motility (Flagella and Type IV Pili)

Flagella are essential for *P. aeruginosa* initial attachment to surfaces. Twitching motility has been necessary for normal biofilm development after microcolony formation [80]. The flagellum is also responsible for *P. aeruginosa* swimming motility in low-viscosity environments. This process occurs through rotation, producing a force moving the bacterium forward [81]. Flagellar attachment is important in initial biofilm establishment, whereas the motility mechanism is associated with dispersal in the final biofilm steps. Robust biofilms during the maturation stage requires an adequate timing of motility control [82].

Twitching motility depends on type IV pili, which play an important role in mediating adherence to mucosal surfaces and subsequent colonization [83]. The retraction and extension of type IV pili, which propel the cells along the surfaces, is linked to the flagellum-independent type of surface motility. [84]. Moreover, these pili are polarly located hair-like filamentous appendages. The structure of pili can be divided as follows subcomplex: (i) PilBTUCD (the cytoplasmic motor), (ii) PilMNOP (the inner membrane alignment), and (iii) PilQF (OM secretin pore) [85].

As they mediate motility and adhesion, pili are considered crucial elements for initiating infections. Additionally, these elements control twitching motility, which is used for prompt colonization of different surfaces [86], implying that sequential cycles of extension, adhesion, and retraction of T4P fibers generate the force to drive the bacterial cell forward [87]. The pilus movement occurs through two cytoplasmic membrane-associated ATPases, which, respectively, polymerize and depolymerize PilA [86]. Moreover, natural transformation and biofilm formation are probably associated with the binding of pili tips to DNA [88].

### 7.1. Immune Evasion (Elastase and Alkaline Protease)

LasA and LasB elastases, type IV protease (PIV), *P. aeruginosa* small protease (PASP), Large ExoProtease A (LepA), alkaline protease (AprA), *P. aeruginosa* aminopeptidase (PAAP), and MucD are extracellular proteases associated with critical invasion in acute infection caused by *P. aeruginosa* [89].

LasB and LasA elastases are secreted by T2SS regulated by QS systems degrading host elastin [90]. LasB elastase is a metalloprotease and the most abundant protease, the main extracellular virulence factor and is encoded by the lasB gene. Recently, antibiotic resistance in clinical isolates of *P. aeruginosa* has been correlated with LasA expression.

Alkaline protease, which is called aeruginolysin in *P. aeruginosa*, another metalloendopeptidase produced through T1SS (aprA gene encoded) [91] which interferes with endothelial components (fibronectin and laminin) and degrades cytokines (IFN, TNF, and IL-6) and complement proteins (C1q, C2, and C3), allows phagocytic evasion. It also cleaves free flagellin monomers, reducing the mucociliary clearance of bacteria through epithelial sodium channel activation [92,93,94], and contributes to the production of pyocyanin (and other virulence factors) [95].

### 7.2. Antibiotic Resistance (Pump Efflux and Modifying Enzymes)

β-lactamases production has been the most important antimicrobial resistance mechanism, once β-lactams are the first line therapy, including piperacillin, ceftazidime, cefepime, ceftolozane, carbapenem, and others [96]. The most important β-lactamases in *P. aeruginosa* are cephalosporinases, ampC hyper-expression, extended-spectrum β-lactamases, and carbapenemases. Currently, carbapenemases has been the major challenge, reducing therapeutic options to just a few, including old and toxic drugs (polymyxins and aminoglycosides) [97,98,99,100,101,102].

The efflux pump systems, responsible for expelling antibiotics out of the cell, are among the best described resistance features [103]. It is important to highlight that drug-resistant *P. aeruginosa* has become an international problem. Efflux pumps have been reported to contribute to the highest level of resistance in synchronization with bacterial biofilms and outer membrane barriers [104,105,106,107]. In this mechanism, the crucial cause of antibiotic multidrug resistance is associated with efflux transporters [108].

The high-level resistance in numerous bacterial species is result of enzymatic modifications of aminoglycosides. Among the important resistance genes transferable between Gram-negative bacteria, mainly in *P. aeruginosa*, are the aminoglycoside-modifying enzyme (AME)-encoding genes [109]. Aminoglycosides are considered the most powerful drugs of choice for the treatment of life-threatening infections caused by *P. aeruginosa*. However, enzymatic modification is one of the most important mechanisms that lead to resistance against them [110].

AMEs are classified as acetyltransferases (AACs), nucleotidyltransferases (ANTs), and phosphotransferases (APH) [111], which initiate N-acetylation, O-nucleotidylation, and/or O-phosphorylation of the aminoglycosides, respectively, resulting in the inactivation of drugs and therapeutic ineffectiveness [111,112]. Moreover, mobile genetic elements (integrons, plasmids, and transposons) can assist in the prompt spreading of clinically important genes [113]. Further, a group linked the main cause for resistance to aminoglycosides in MDR isolates of *P. aeruginosa* to the presence of AME genes [114].

### 7.3. Cytotoxicity (HCN, Exotoxin A, T3SS, and Pyocyanin)

Only a few bacterial species such as *P. aeruginosa* synthesize HCN. Cyanide is a secondary metabolite that can inhibit many cellular processes; it has been reported that HCN inhibits aerobic respiration [115]. HCN is detectable in the headspace of in vitro *P. aeruginosa* cultures [116,117] and can be found in samples of sputum of cystic fibrosis patients infected with *P. aeruginosa* [118].

Exotoxin A is classified as the major *P. aeruginosa* toxigenic virulence factor exocyted through T2SS by most of the isolates [119]. It is divided into three structural domains and one subdomain. The N-terminal domain (I), responsible for attachment to host cells, is composed primarily of antiparallel β-strands; the middle domain (II) is composed of six α-helices with membrane translocating activity; and the C-terminal domain (III) is the toxic moiety [120].

The *P. aeruginosa* T3SS, responsible for injecting toxic effectors into the host cell cytosol (injectisome), is composed of several proteins, including (i) a secretion apparatus (transmembrane transports effectors) and (ii) a translocation apparatus (moves the effectors through human cell membranes) [121]. The secretion apparatus has a helically polymerized protein (PscF) and a cytoplasmic ATPase (PscN), an IM lipoprotein ring (PscJ), and an oligomerized secretin ring at the OM (PscC). Two hydrophobic proteins (PopB and PopD) comprehend the translocation apparatus, interacting with the human cells membrane. They form a pore of translocation, and PcrV (a hydrophilic protein), which are necessary for the perfect assembly and insertion of PopB and PopD into human cell surface [121].

T3SS is not required for infection, however, it enhances the infection severity, enabling *P. aeruginosa* to cause injury of the epithelium, and disseminating into the circulation and counteracting host innate immune responses (through an effector-independent or dependent pathway) [121].

The blue-greenish color of *P. aeruginosa* colonies in culture can be related to pyocyanin, which is a redox-active secondary metabolite [91]. The disease severity and lung function decline due to this phenazine secreted by T2SS result from its free radical and pro-inflammatory effects [122]. It can increase intracellular ROS and H_2_O_2_ levels, inducing oxidative stress and damaging several enzymes, components of the cell cycle, and DNA; this leads to cell lysis [122]. As a result, eDNA is released, and this may contribute to biofilm formation and the persistence of infections [123]. Mitochondrial ROS release is also induced, leading to neutrophil apoptosis [124]. In addition, pyocyanin causes epithelial disruption by slowing the ciliary beating. It also increases respiratory mucous production, contributing to lung colonization. This phenomenon also increases IL-8 synthesis by alveolar macrophages [122].

### 7.4. Iron Scavenging (Proteases and Siderophores)

Pyochelin is a siderophore based on salicylate with a low iron affinity, whereas pyoverdine (PVD) is a peptide [125]. The process of PVD production consumes energy in *P. aeruginosa*, which primarily produces pyochelin, and switches to PVD production only when the iron concentration becomes really low [126]. PVD comprises a binding iron conserved dihydroxyquinoline chromophore. It is reported that more than fifty pyoverdines are produced, but they are classified into three types based on the variation in the peptide chain (PVDI, PVDII, and PVDIII) [125]. One of the pyoverdine (PVD) scavenges iron from human proteins accomplished through a membrane network as well as efflux-pumps and transporters in the periplasmic space [127]. Although essential, high concentration of iron produces reactive oxygen species, which leads to cytotoxic effect in the cells [128]. Therefore, this system is shut off by the ferric uptake regulator (called Fur) when sufficient intracellular iron is present [129]. The ECM is important to maintain balance in biofilms, since it stores the iron sequestered within the three EPSs (29948830). PVD acts as a signaling molecule to produce ETA, an endoproteinase (PrpL), and PVD itself.

## 8. *Pseudomonas aeruginosa* Biofilm Treatment

Owing to increasing antibiotic resistance and the extensive spread of associated infections, treatment failure has become a major global concern; *P. aeruginosa* has shown great intrinsic resistance to a range of antibiotics, such as beta-lactams, fluoroquinolones, and aminoglycosides, resulting in relevant morbidity and mortality rates [130]. The most common treatment for *P. aeruginosa* infection is therapy with beta-lactams, whereas carbapenems are the last safe therapeutic alternatives for many MDR infections.

The low antibiotic permeability of the outer membrane (chromosomally encoded AmpC), and drug efflux via multidrug efflux (Mex) systems are the major factors of resistance [131]. *Pseudomonas aeruginosa* has different mechanisms for resistance to various antibiotics, such as horizontal gene transfer and mutation-driven resistance [130,132]. It has been highlighted that MDR *P. aeruginosa* acquire mobile genetic elements (transposons, resistance islands, prophages, integrons, and plasmids) through transmission and accommodate them in antibiotic resistance genes [133]. Some of the consequences of this are biofilm formation and the prevalence of inherent and acquired antibiotic resistance of *P. aeruginosa* in recent years. These have led to a situation where there are few effective antibiotics available to stop this bacterium [134]. Additionally, it has been noted that the minimum inhibitory concentrations (MICs) of bacteria inside biofilms may be 10–10,000 times higher than that of planktonic cells [135,136,137].

## 9. Therapeutics for *Pseudomonas aeruginosa* Infections

*Pseudomonas aeruginosa* infections are a great concern as they make the clinical use of regular antimicrobials challenging for therapeutic management. In addition, *P. aeruginosa* is known to present multiple drug tolerance apparatus, which can be divided as follows: (i) intrinsic, (ii) acquired, and (iii) adaptive mechanisms. It is important to point out that biofilm formation is an adaptive mechanism. Survival under exposure to antibiotics and the initiation of chronic infections are crucially associated with this mechanism, which is a virulence factor enhancer [130]. Multifactorial processes are associated with the development and dispersal of biofilms, which require QS systems, c-di-GMP and exopolysaccharides [138]. Hence, reversing strategies are needed to achieve different constituents of the biofilm-residing cells and matrix [139].

Furthermore, it is crucial to ponder the interplay among the host immune system and the bacteria when managing biofilm-related infections [140,141]. Essentially, chronic infections are commonly polymicrobial, which complicate the patient outcomes compared to monomicrobial infections. This scenario has significantly influenced the biofilm therapeutic strategies aimed at disrupting the biofilm communities of mono-species [142,143]. Inducing biofilm dispersal, inhibiting QS, and targeting iron metabolism using different antimicrobial agents, including antimicrobial peptides, QS inhibitors, biofilm-degrading enzymes, and iron chelators, are some strategies that have been evaluated to overcome these issues. A promising alternative to combat the pathogenicity of *P. aeruginosa* is the use of human probiotic bacteria, such as *Lactobacillus casei* CRL 431 and *Lactobacillus acidophilus* CRL 730, with the intention of mitigating *P. aeruginosa* biofilm strength and virulence by QS inhibition through metabolites [144].

## 10. Therapeutic Options for *Pseudomonas aeruginosa* Infections

Research on antimicrobials is focused on exploring new approaches and therapies beyond conventional antibiotics, such as bacteriophages, antimicrobial peptides with diverse structures and mechanisms of action, virulence inhibitors, siderophores, compounds of natural origin (e.g., oils), and other adjuvants (e.g., efflux pump inhibitors, monoclonal antibodies), owing to the astounding increase in antimicrobial resistance rates in all types of bacteria [134,145,146].

Researchers have been investigating new methods to inhibit *P. aeruginosa* biofilms. Recently, some studies have indicated that phage therapy is an effective approach for destroying *P. aeruginosa* biofilms [147,148]. Other novel methods aimed at suppressing the biofilms formed by *P. aeruginosa*, such as small molecule-based inhibitors, phytochemicals, bacteriophage therapy, photodynamic therapy, antimicrobial peptides, monoclonal antibodies, and nanoparticles, are being considered [149].

Medical device-related biofilm infections are a great concern. The development of materials resistant to biofilm colonization is a great strategy to prevent such infections. Some strategies to make materials less susceptible to biofilm attachment involve impregnation of devices with antimicrobial compounds, use of nanoparticle-embedded materials, or modulation of the nanostructure of materials [150].

An example of the impregnation of devices is the silver coating of endotracheal tubes (ETTs), which has demonstrated efficacy against *P. aeruginosa* infection. In an experimental canine model of *P. aeruginosa* VAP, an ETT coated with an antimicrobial silver hydrogel coating underwent notably less *P. aeruginosa* colonization and inflammation than an uncoated ETT [151]. In another large, prospective, single-blind, randomized, controlled study, it was noticed that patients who received a silver-coated ETT exhibited a significant reduction in the incidence of VAP and recurrence compared with those who received an uncoated tube [152], suggesting that antibacterial coating may prevent biofilm developing and reduce VAP incidence.

In addition, a research group found that the strategy of using bacteriophage cocktail-coated tubes had the potential to control the ETT-associated *P. aeruginosa* biofilms [153].

Newer approaches to inhibit *P. aeruginosa* QS signaling are being sought. To this end, halogenated furanones (HF) of macroalga *Delisea pulchra* were the first bacterial QS inhibitors identified. Despite not demonstrating activity against *P. aeruginosa* QS, synthetic furanones have been developed. A variety of synthetic HF compounds have been evaluated and demonstrated biofilm biomass reduction, increase the susceptibility of *P. aeruginosa* biofilms to tobramycin in vitro. Moreover, this approach decreased the pathogenicity of *P. aeruginosa* in vivo in mouse pulmonary infection models [154,155]. The development of novel synthetic furanones through a variety of biochemical screens is currently the focus of attention [156,157].

Diverse natural compounds (and their derivatives) that can inhibit QS have been investigated. For instance, an increase in the antimicrobial susceptibility of *P. aeruginosa* and a decrease in the pathogenicity were observed upon the application of garlic extract, specifically ajoene, the chemical found in garlic, which inhibited QS-mediated virulence factors [158,159]. In contrast, a clinical study using garlic in patients with cystic fibrosis and chronic *P. aeruginosa* infection showed a significant difference compared with the controls [160]. Patulin (a metabolite produced by *Penicillium* spp.) decreased *P. aeruginosa* virulence in vitro, resulting in increased bacterial clearance in a mouse pneumonia model [161]. Several other natural compounds have also been evaluated [162,163] using in vitro systems, including iberin, found in horseradish [162], eugenol, found in clove extract [163], and ellagic acid from *Terminalia chebula* fruit [15]. However, further studies are necessary to investigate these compounds using preclinical models.

QS inhibition effects have been evaluated using azithromycin and erythromycin [164,165]. For instance, a study using azithromycin showed decreased inflammation in a mouse model of chronic *P. aeruginosa* pulmonary infection [166]. In addition, individuals with cystic fibrosis and *P. aeruginosa* infection showed improved clinical outcomes when using azithromycin [167].

Another strategy for QS inhibition is therapy using quorum-quenching enzymes, for instance, acylases and lactonases. These degrade AHLs, or oxireductases, which inactivate AHLs via modification [168]. A reduction in lung injury and mortality from 75% to 20% in a rat *P. aeruginosa* model was observed upon nasal administration of the lactonase, SsoPox-1, which attenuated QS signaling, virulence factor production, and biofilm formation in vitro [168]. In nematode infection models and in vitro systems using *P. aeruginosa*, it was demonstrated that AHL acylases, such as AiiD acylase and PvdQ, and the oxidoreductase Bpi09 also produce favorable results [169,170,171].

Enzymes with lactonase activity that are expressed by host cells are classified as PONs. These enzymes, mainly PON-2, are capable of hydrolyzing AHL molecules and thereby inhibiting QS and downstream virulence and biofilm formation [172,173]. It has been reported that mice deficient in PON exhibit a marked impairment in their ability to hydrolyze 3-oxo-C12-HSL and clear *P. aeruginosa* [174]. In patients with cystic fibrosis, lower incidence of PON-2 expression was associated with a higher incidence of *P. aeruginosa* infections [175]. In addition, it was pointed out that *P. aeruginosa* has the ability to directly modulate the host response by attenuating PON-2, which is mediated by the QS molecule 3-oxo-C12-HSL [176,177].

Novel therapies against biofilms, such as the suppression of c-di-GMP signaling, have been proposed owing to the importance of c-di-GMP signaling in biofilm formation and dispersal. This was evaluated through a study, which used a foreign body infection model of *P. aeruginosa*. It was observed that the levels of c-di-GMP could be reduced by inducing a phosphodiesterase. Moreover, the attenuation of bacterial c-di-GMP signaling enabled greater clearance of the infection [178]. However, a study using doxorubicin (previously identified as a potent c-di-GMP inhibitor in a large screen) paradoxically showed an increase in biofilm size [179]. Despite these advances, further studies are necessary to ascertain whether suppression of c-di-GMP signaling is a potential therapy.

Organisms from all classes of life produce peptides with antimicrobial properties, and several synthetic antimicrobial peptides have been developed [180]. Some of these peptides decrease the attachment of bacterial cells, preventing bacterial biofilm formation [181]. Optimistic findings were obtained when analyzing these peptides in in vitro systems. However, further studies are necessary before these discoveries can be translated into clinical studies [182,183,184].

## 11. Conclusions

The pathogenicity mechanisms of *P. aeruginosa* biofilms, including their characteristics and QS properties, are extremely complex. Although the structure of a biofilm seems to be simple, the genes and mechanisms involved in biofilm formation are diverse and hard to comprehend. Furthermore, most of the studies to identify them have been conducted in vitro, which is not the real situation in human infections. The use of transcriptomics and metabolomics to analyze clinical sample could be an approach to address this. However, it would be difficult to separate the results pertaining to *P. aeruginosa* from those of other bacteria, since most of these infections are polymicrobial and involve local microbiota. It is important to elucidate the interaction of *P. aeruginosa* with the host for the further development of new therapies.

## Figures and Tables

**Table 1:** Virulence mechanisms employed during *Pseudomonas aeruginosa* infections.

Antibiotic resistance	Efflux pumps	
	Modifying enzymes	
Motility	flagella	
	Type IV pili	
Biofilm structure & dynamics	Rhamnolipids	
	alginate	
Iron scavenging	proteases	
	siderophores	pyochelin
		pyoverdine
Cytotoxicity	pyocyanin	
	T3SS	
	Endotoxin A	
	HCN	
Immune evasion	elastase	
	Alkaline protease	

## Data Availability

Not applicable.
